# Intercalating and maintenance gefitinib plus chemotherapy versus chemotherapy alone in selected advanced non-small cell lung cancer with unknown *EGFR* status

**DOI:** 10.1038/s41598-017-08399-8

**Published:** 2017-08-16

**Authors:** Hong Jian, Wei Li, Zhiyong Ma, Jianjin Huang, Jifeng Feng, Yong Song, Beili Gao, Huili Zhu, Min Tao, Chong Bai, Shenglin Ma, Hongming Pan, Shukui Qin, Dong Hua, Yongfeng Yu, Shun Lu

**Affiliations:** 1 Shanghai Lung Cancer Center, Shanghai Chest Hospital, Shanghai Jiao Tong University, Shanghai, China; 2 Cancer Center, the First Hospital of Jilin University, Changchun, China; 3 Department of Internal Medicine, Henan Cancer Hospital, Zhengzhou, China; 4 Department of Medical Oncology, The Second Affiliated Hospital of Zhejiang University School of Medicine, Hangzhou, China; 5 Department of Medical Oncology, Jiangsu Cancer Hospital, Nanjing, China; 6 Department of Respiratory Medicine, Nanjing General Hospital of Nanjing Military Command, Nanjing, China; 7 Department of Respiratory Medicine, Ruijin Hospital, Shanghai Jiao Tong University School of Medicine, Shanghai, China; 8 Department of Respiratory Medicine, Huadong Hospital Affiliated to Fudan University, Shanghai, China; 9 Department of Oncology, The First Affiliated Hospital of Soochow University, Soochow, China; 10 Department of Respiratory Medicine, Changhai Hospital Affiliated to Shanghai Second Military Medical University, Shanghai, China; 11 Department of Radiotherapy, Hangzhou First People’s Hospital, Hangzhou, China; 12 Department of Medical Oncology, Sir Run Run Shaw Hospital, School of Medicine, Zhejiang University, Hangzhou, China; 13 Department of Medical Oncology, PLA 81 Hospital, Nanjing, China; 14 Department of Medical Oncology, Wuxi Fourth People’s Hospital, Wuxi, China

## Abstract

Epidermal growth factor receptor tyrosine-kinase inhibitors (EGFR-TKIs) are standard treatment for advanced non-small cell lung cancer (NSCLC) patients with epidermal growth factor receptor (EGFR) mutation. However, *EGFR* mutation testing is not attainable in approximately 20% of patients. The current study examined intercalating and maintaining gefitinib treatment in stage IIIB/IV non-squamous NSCLC, never or former light smoking patients with unknown *EGFR* mutation status. Briefly, 219 patients who achieved stable disease (SD) with gemcitabine (1250 mg/m^2^) plus carboplatin (5 AUC) were randomized at 1:1 ratio to continue chemotherapy (n = 110) or intercalating gefitinib (250 mg/day on days 15–25 of each cycle until disease progress (n = 109). Progression-free survival (PFS) was 9.7 vs. 4.2 month in the gefitinib vs. control arm (HR: 0.41, 95% CI: 0.31–0.56; *P* < 0.001). Overall survival (OS) was also longer in the gefitinib arm (20.1 vs. 15.4 months; HR: 0.68; 95% CI 0.48–0.97; *P* = 0.0323). Adverse events, including diarrhea, dermal reaction and thrombocytopenia, were more common in the gefitinib arm. In conclusion, intercalating and maintenance gefitinib treatment is a viable option for advanced NSCLC patients with unknown *EGFR* mutation status in subpopulations with high EFGR mutation rate.

## Introduction

Lung cancer, mostly non-small cell lung cancer (NSCLC), is a leading cause of cancer death worldwide^1^. Surgical resection is the only chance for cure. However, NSCLC is non-resectable in majority of the patients upon diagnosis.

Molecular events that drive the development of NSCLC include epidermal growth factor receptor (*EGFR*) mutations and anaplastic lymphoma kinase (*ALK*) gene rearrangements. These discoveries have led to targeted therapies^2–4^. The Iressa Pan Asian Study (IPASS) demonstrated superiority of EGFR tyrosine kinase inhibitors (EGFR-TKIs) to conventional chemotherapy in prolonging progression-free survival (PFS) and increasing objective response rate (ORR) in NSCLC patients harbouring *EGFR-*sensitive mutations^5^. This finding has been confirmed by a number of phase III trials in advanced NSCLC patients with *EGFR* mutation^6–12^.

Several major lung cancer treatment guidelines recommend that lung cancer patients be screened for *EGFR* mutations and advanced NSCLC patients harbouring *EGFR-*sensitive mutations be given first line TKI therapy whereas patients with negative or unknown *EGFR* mutation status be given platinum-based regimens^13, 14^. *EGFR* mutation status is the most reliable predictor of clinical response to EGFR-TKIs. However, despite availability of massive, parallel-sequencing technologies enabling efficient, simultaneous detection of driver mutations in lung cancer^15, 16^, up-front assessment of *EGFR* mutation status in all patients with advanced NSCLC is not attainable for a number of reasons, including non-evaluable/unavailable samples (in approximately 20% of the samples) and lack of access to affordable testing technologies, particularly in the Asia-Pacific region and other less developed regions of the world^17^. For this subset of patients, we speculate that intercalating EGFR-TKI into chemotherapy could provide some benefits.

The TRIBUTE study examined erlotinib (150 mg/d) vs placebo in combination with up to six cycles of paclitaxel and carboplatin, followed by maintenance monotherapy for advanced NSCLC. The overall analysis failed to show a benefit with erlotinib but subgroup analysis indicated survival benefit in never smokers^18^. In the FAST-ACT1 and FAST-ACT2 trials, naïve patients with advanced NSCLC received either erlotinib (150 mg/d) or placebo on days 15–28 of a 4-week cycle that included gemcitabine plus either cisplatin or carboplatin^19, 20^. FAST-ACT1 revealed longer progression-free survival (PFS), whereas the FAST-ACT2 showed longer overall survival (OS) with erlotinib intercalated in chemotherapy. Of the 451 patients in the FAST-ACT2 study, analysis of the 256 subjects with biomarker analysis information revealed that activating EGFR mutations are predictor for improved treatment outcome^21^.

EGFR mutation is more common in certain patient subpopulation, including adenocarcinoma, East Asian ethnicity, females and never smokers; also these subjects tend to respond better to EGFR-TKIs^22^. In the current, we compared intercalating and maintenance use of gefitinib plus chemotherapy (vs. chemotherapy alone) in selected advanced NSCLC patients with unknown *EGFR* status. The trial was limited to subjects who had achieved stable disease (SD) following two cycles of chemotherapy: those who developed progressive disease (PD) are clearly not suitable candidates for planned intervention whereas there is no reason to change the treatment in those who achieved partial remission (PR). The selected subgroup was also limited to non-squamous NSCLC in never smokers or former light smokers since mutation is particularly high in this subpopulation and therefore the intervention is more likely to produce sizable benefits.

## Results

### Patient Demographic and Baseline Characteristics

**Figure 1:**
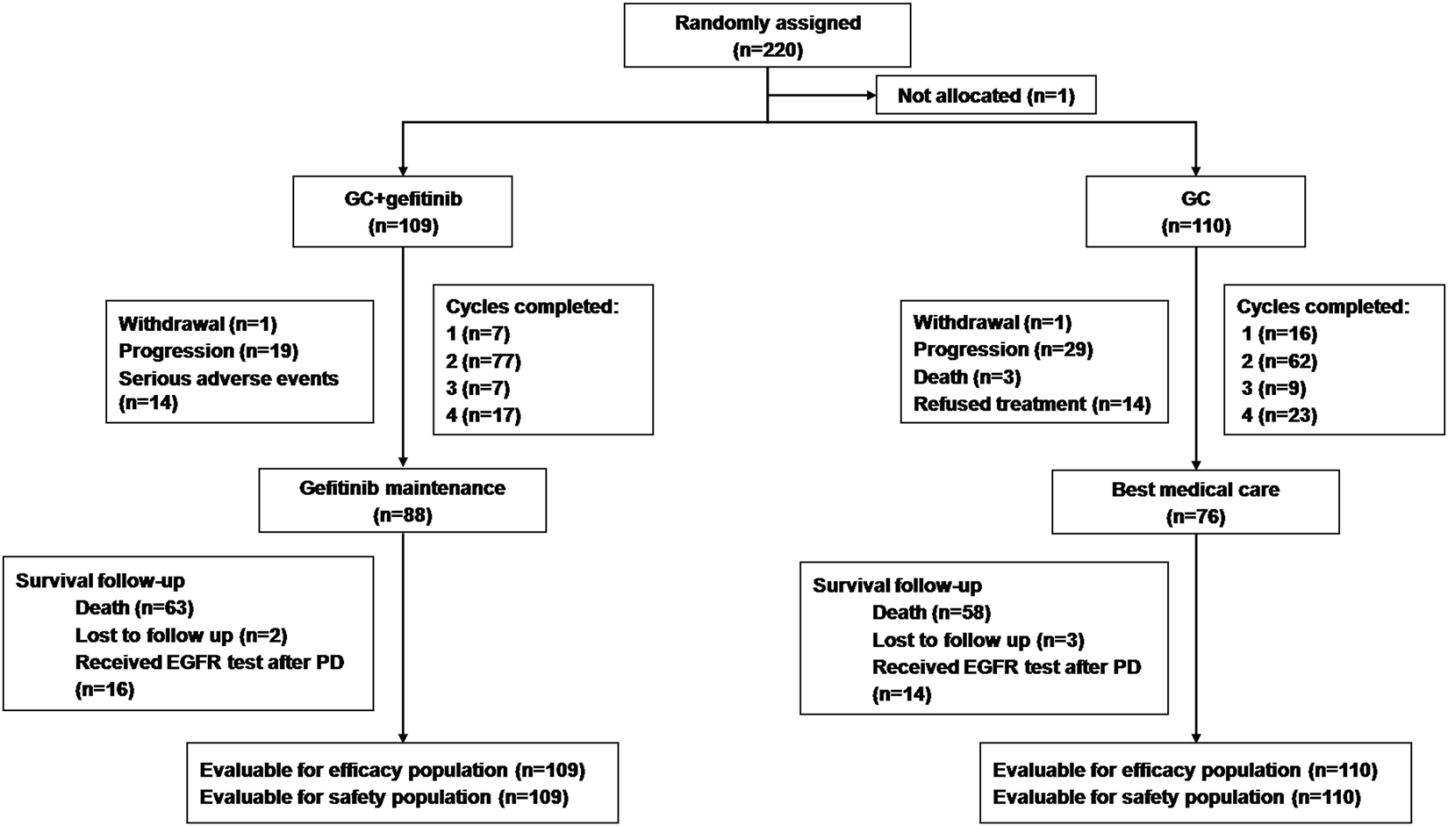
The study flow chart. GC = gemcitabine; PD = progressive disease.

Between June 2011 and September 2014, 220 eligible subjects were randomized to a control (n = 110) or gefitinib arm (n = 109, 1 patient withdrew without any treatment) (Fig. 1). Patients in the two arms were comparable in demographic and baseline characteristics (Table 1).

**Table 1:** Baseline Characteristics of the 2 Arms.

Characteristic	Arm A	Arm B	
No. of patients	109	110	
Age, years			0.7266
Median	56.0	58.0	
Range	49–62	50–63	
Female gender, n(%)	88 (80.7)	83 (75.5)	0.345
Smoking status, n(%)			1
Former smoker	1(0.9)	1(0.9)	
Never smoker	108 (99.1)	109 (99.1)	
Pathology, n(%)			0.2123
Adenocarcinoma	105 (96.3)	109 (99.1)	
Adenosquamous carcinoma	4 (3.7)	1 (0.9)	
ECOG performance status, n(%)			0.6278
0	35 (32.1)	32 (29.1)	
1	74 (67.9)	78 (70.9)	
Current disease stage, n(%)*			0.3079
IIIB	6 (5.5)	10 (9.1)	
IV	103 (94.5)	100 (90.9)	
Brain metastasis			0.1716
Yes	16 (14.7)	24 (21.8)	

NOTE: The ITT dataset was used to generate this table. Arm A: gemcitabine + carboplatin + gefitinib; Arm B: gemcitabine + carboplatin. *American Joint Committee on Cancer (AJCC) stage. Abbreviation: ECOG = Eastern Cooperative Oncology Group.

### Treatment Characteristics

The gefitinib and control arms received a mean of 2.3 and 2.4 cycles of chemotherapy, respectively; 15.6% in the gefitinib arm and 20.9% in the control arm completed four cycles of chemotherapy. Dose reduction was required in 47 (43.1%) patients in the gefitinib arm and 47 (42.7%) patients in the control arm. One (0.92%) patient in the gefitinib arm and 8 (7.3%) patients in the control arm experienced treatment delay. 22 (20.2%) patients in the gefitinib arm and 32 (29.1%) patients in the control arm experienced treatment interruption.

### Efficacy

**Figure 2:**
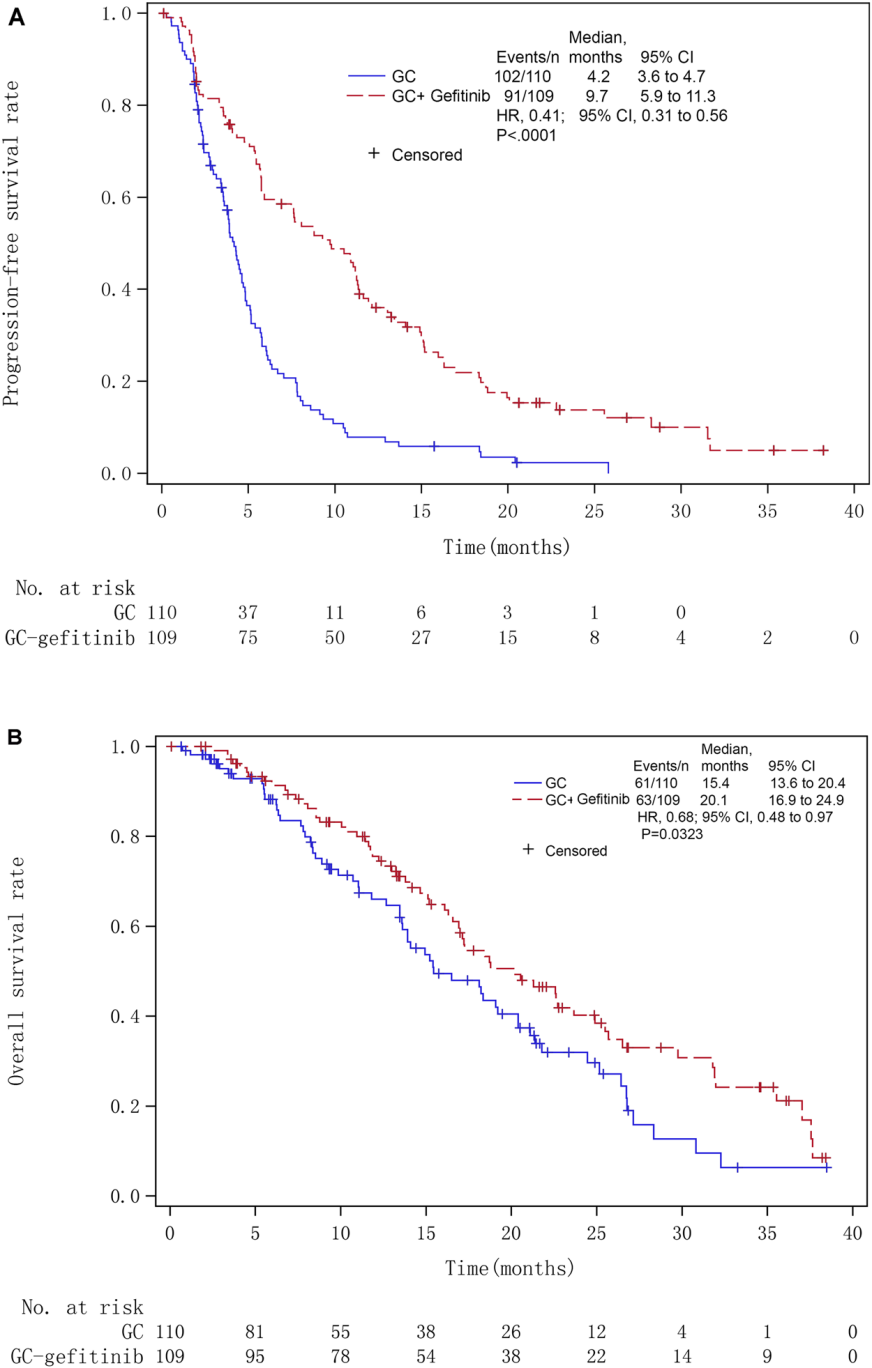
Kaplan-Meier curve for (**A**) progression-free survival (PFS) and (**B**) overall survival (OS) of intention-to-treat non-small cell lung cancer (NSCLC) patients receiving intercalating and maintenance use of gefitinib plus chemotherapy vs. chemotherapy alone. GC = gemcitabine; HR = hazards ratio.

The median follow-up duration was 20.5 months (range, 0.7–49.7). The median PFS in the gefitinib arm (9.7 months; 95% CI, 5.9–11.3; 91 events) was significantly higher than in the control arm (4.2 months; 95% CI, 3.6–4.7; 201 events; *P* < 0.0001) (Fig. 2A). The HR was 0.41 (95% CI: 0.31–0.56; *P* < 0.001).

Sixty-three (57.8%) patients in the gefitinib arm and 61 (55.5%) patients in the control arm died. The median OS in the gefitinib arm (20.1 months; 95% CI, 16.9–24.9) was significantly longer than in the control arm (15.4 months; 95% CI, 13.6–20.4; HR: 0.68; 95% CI, 0.48–0.97; *P* = 0.0323) (Fig. 2B).

Table 2 shows the overall responses at 8 weeks after randomization. No patient in either arm achieved CR. Twenty-one (19.3%) patients in the gefitinib arm and one (0.9%) in the control arm achieved PR. Sixty-eight (62.4%) patients in the gefitinib arm and 79 (71.8%) patients in the control arm achieved SD. Seventeen (15.6%) patients in the gefitinib arm and 21 (19.1%) patients in the control arm showed progression. The ORR of the gefitinib arm (19.3%) was significantly higher than in the control arm (0.9%) (*P* < 0.0001).

**Table 2:** Overall Response at 8 Weeks after Randomization according to RECIST 1.0.

Parameters	Arm A (N = 109)	Arm B (N = 110)	*P* value
Best overall response, n(%)			
Complete response	0	0	<0.0001^
Partial response	21 (19.3)	1 (0.9)	
Stable disease	68 (62.4)	79 (71.8)	
Progressive disease	17 (15.6)	21 (19.1)	
Not evaluable	3 (2.8)	9 (8.2)	
ORR	21 (19.3)*	1 (0.9)	<0.0001^

NOTE: Objective response rate (ORR) = Complete response plus partial response. Arm A: gemcitabine + carboplatin + gefitinib; Arm B: gemcitabine + carboplatin. *Calculated by simple ratio; ^Fisher’s exact test.

### Efficacy Stratified by EGFR Mutational Status

*EGFR* mutation status became available during the course of this study in a subset of patients (n = 30): 17 (56.7%, 95% CI, 38.9–74.4%) patients had *EGFR* sensitive mutations. The 7 subjects with *EGFR* sensitive mutations in the gefitinib arm (n = 7) had longer median PFS (12.1 months, 95% CI, 4.0–18.7) vs. those in the control arm (n = 10; 3.9 months, 95% CI, 2.0–4.6) (Table 3). In contrast, the PFS did not differ between subjects with wildtype *EGFR* receiving gefitinib vs. control (gefitinib, n = 9; median PFS, 3.4 months, 95% CI, 1.7–9.3 vs. controls, n = 4; median PFS, 5.0 months, 95% CI, 2.0–7.8). In patients with *EGFR-*sensitive mutations, the OS was also longer in the gefitinib arm (32.0 months, 95% CI, 10.9–35.5 vs. 21.1 months, 95% CI, 11.1–32.3). In subjects with *EGFR-*sensitive mutations, objective response was achieved in 2 patients (28.6%, 2/7) in the gefitinib arm, but none in the control group. Furthermore, no patients with wildtype *EGFR* in either arm showed objective response.

**Table 3:** Efficacy of Intercalating and Maintenance Use of Gefitinib plus Chemotherapy vs. Chemotherapy alone for NSCLC Patients Stratified by *EGFR* Mutational Status (n = 30).

	Sensitive *EGFR* mutations		Wildtype *EGFR*	
	Arm A	Arm B	Arm A	Arm B
No. of patients	7	10	9	4
ORR	2	0	0	0
Median PFS (months)	12.1 (95% CI, 4.0–18.7)	3.9 (95% CI, 2.0–4.6)	3.4 (95% CI, 1.7–9.3)	5.0 (95% CI, 2.0–7.8)
Median OS (months)	32.0 (95% CI, 10.9–35.5)	21.1 (95% CI, 11.1–32.3)	16.9 (NA)	NA

NOTE: Objective response rate (ORR) = Complete response plus partial response; Arm A: gemcitabine + carboplatin + gefitinib; Arm B: gemcitabine + carboplatin. Abbreviation: HR = hazards ratio; OS = overall survival; PFS = progression-free survival.

### Safety

Safety data are summarized in Table 4. Leukopenia was the most common AE in both arms (control, 80.9% vs. gefitinib, 79.8%). Significantly more patients in the gefitinib arm had diarrhea (12.8% vs. 0.9% in the control) and skin rashes (8.3% vs. 0.9% in the control); *P* < 0.01). Approximately half of the patients in the control and gefitinib arm experienced an AE of CTCAE grade >3 (Table 4). Significantly more patients in the gefitinib arm (37.6%) had thrombocytopenia than in the control arm (23.6%) (*P* = 0.03). Treatment-emergent AEs that led to treatment discontinuation was not noted. No increase in gefitinib-emergent interstitial lung disease was noted.

**Table 4:** Adverse Events [n(%)].

	Arm A (N = 109)	Arm B (N = 110)	*P* value
Common adverse events (any grade)			
Leukopenia	87(79.8%)	89(80.9%)	0.8387
Neutropenia	73(67.0%)	75(68.2%)	0.8484
Thrombocytopenia	62(56.9%)	53(48.2%)	0.1974
Anemia	76(69.7%)	71(64.5%)	0.4146
Abnormal ALT	14(12.8%)	15(13.6%)	0.8627
Abnormal plasma creatinine	14(12.8%)	10(9.1%)	0.3740
Nausea	23(21.1%)	13(11.8%)	0.0638
Vomiting	14(12.8%)	9(8.2%)	0.2605
Diarrhea	9(8.3%)	1(0.9%)	0.0098
Dermal and subcutaneous disease	9(8.3%)	1(0.9%)	0.0098
Adverse Events (Frequency of ≥10%*) of CTCAE grade > 3			
All AEs, n(%)	59(54.1%)	55(50.0%)	0.5409
Leukopenia	17(15.6%)	26(23.6%)	0.1342
Neutropenia	28(25.7%)	25(22.7%)	0.6090
Anemia	15(13.8%)	9(8.2%)	0.1863
Thrombocytopenia	41(37.6%)	26(23.6%)	0.0248

NOTE: Arm A: gemcitabine + carboplatin + gefitinib; Arm B: gemcitabine + carboplatin. *Frequency >10%: authors only chose to report an CTCAE (grade > 3) if it occurs in > 10% of the population.

## Discussion

This multicenter study found that intercalating and maintenance use of gefitinib significantly prolonged PFS and OS of selected group of patients with advanced NSCLC and unknown *EGFR* status who had achieved SD on chemotherapy. After two courses of gefitinib therapy, the ORR of the gefitinib arm was higher than that in the control arm. AEs in the getitinib arm in the current study included thrombocytopenian, diarrhea and skin rashes, but considering previously reported rate, were generally within the accepted range.

The study lends strong support to the finding from FAST-ACT2 that intercalating erlotinib into chemotherapy could prolong PFS and OS. The HR for PFS was 0.41 (95% CI 0.31–0.56) in our study vs. 0.57 (95% CI 0.47–0.69) in FAST-ACT2 and the HR for OS was 0.68 (95% CI 0.48–0.97) in our study vs. 0.79 (95% CI 0.64–0.99) in FAST-ACT2. The more benefits of PFS and OS may come from following reasons, firstly, in our study, subjects were Asians and had non-squamous NSCLC patients; all were non-smokers or formerly light smokers. Previous studies showed high *EGFR* mutational rate with these features, even in Caucasians^23–26^. In the current study, *EGFR* mutation analysis was carried out in 30 patients after subjects entered the study; the results revealed an *EGFR* mutation rate of 56.7%. These patients represent a subset of patients who likely respond to EGFR-TKIs. Secondly, unlike FAST-ACT2, our patients received two cycles of platinum-based regimen prior to randomisation. Those who achieved SD went on to receive intercalating use of gefitinib whereas those who achieved CR/PR or developed PD were not given gefitinib. Patients who did not progress during intercalating therapy received gefitinib maintenance therapy. This therapeutic design prevented those patients not responsive to chemotherapy from receiving 2–4 additional cycles of chemotherapy and those insensitive to EGFR-TKIs from receiving EGFR-TKIs maintenance therapy. In addition, we chose to intercalate gefitinib on days 15–25 of chemotherapy to minimize the of entry into the S phase by cancer cells^27^. Day 26–28 was the washout period (FAST-ACT2 had no washout period).

The control arm in the current study was not placebo or first-line standard option for non-squamous NSCLC (platinum-pemetrexed or bevacizumab-based chemotherapy). However, we believe that the effect size (PFS reduction (9.7 to 4.2 months)) could not be reasonably attributed to the bias introduced by such a design and regimens. Importantly, this intercalating use of gefitinib did not increase gefitinib-emergent interstitial lung disease, neutropenia and anemia. The PFS of the current study was seemingly shorter than that reported by other TKI first line efficacy data in *EGFR*-mutated patients^28^, partly because we calculated PFS from the date of randomization when the patients had already received two prior cycles of chemotherapy.

The IPASS showed superiority of gefitinib to chemotherapy in improving PFS of clinically selected patients, but no significant difference in OS because of cross-over and other factors. Other studies also found no difference in OS in *EGFR*-mutated patients receiving first line EGFR-TKI therapy (with first-generation EGFR-TKIs) or chemotherapy. Using a strategy for an intercalating and maintenance use of gefitinib with chemotherapy, we demonstrated that, compared to chemotherapy alone, gefitinib significantly prolonged OS. In a previous meta-analysis of RCTs (6 with erlotinib, 4 with gefitinib), intercalated combination was associated with a significant improvement in overall survival (OS; hazard ratio [HR], 0.82; 95% confidence interval [CI], 0.71–0.95; *P* = 0.01)^29^. The results from the current study supported the notion that intercalating gefitinib in select patients (high probability of *EGFR* mutation but no testing results) is a viable option. We did not observe the responses to treatments subsequent to the compared interventions in our study due to practical difficulty. This is a limitation that requires further study.

The gefitinib arm in our study showed a significant improvement in ORR: 19.3% of patients in the gefitinib arm and 0.9% in the control arm achieved PR; 62.4% of patients in the gefitinib arm and 71.8% of patients in the control arm remained in SD; 15.6% of patients in the gefitinib arm and 19.1% of patients in the control arm progressed. Such results, in our opinion, mostly reflects the lower gefitinib treatment intensity (22-day exposure prior to outcome assessment due to the intercalating nature of the regimen vs. daily gefitinib exposure for >40 consecutive day in previous studies). Another minor factor that may also have contributed to such a difference is the fact that subjects in the current study represented a “selected” subset of patients who did not respond to first-line chemotherapy as opposed to unselected patient groups in previous studies that resulted in higher ORR.

A small number of our patients (n = 30) received *EGFR* testing, and *EGFR* mutated patients receiving gefitinib had longer PFS and RR than patients with *EGFR* mutations receiving chemotherapy alone. However, benefits from intercalating gefitinib were not observed in patient with wild-type *EGFR*. These findings suggest that improved PFS and ORR were at least partially driven by patients harbouring *EGFR* mutations. However, caution should be exercised in interpreting the results given the small size and the retrospective nature of the cohort undergoing *EGFR* testing and a wide 95% CI (56.7%, 95% CI 38.9–74.4%) for positive *EGFR* mutational status. Furthermore, our patients received 2 cycles of chemotherapy in addition to EGFR-TKI in the absence of knowledge of their *EGFR* status. As indicated by retrospective *EGFR* testing, more than 40% of the patients likely experienced unneeded toxicity without benefiting from gefitinib therapy, highlighting the need for *EGFR* testing in NSCLC patients.

Based on current guidelines and practice, treatment decisions of NSCLC are stratified by histology and molecular biomarkers. However, because of undesirable sample quality or lack of access to enabling technologies, *EGFR* status remains unknown in a significant proportion of NSCLC patients in China^30^. In the absence of a molecular predictor, never smoking Asians and patients of the adenocarcinoma subtype could represent a clinically select population with a high likelihood of harboring activating *EGFR* mutations and therefore more likely respond to EGFR-TKI. The clinical significance of the current findings should be viewed in the context in China, where less than 20% of NSCLC patients are tested for *EGFR* mutations while in other Asian countries, 30%-80% of NSCLC patients are tested for *EGFR* mutations^31^. Moreover, *EGFR* mutation test results were available before administration of first line therapy in 77% of patients who were tested, with significant differences between countries (range: 51% in France to 89% in Japan; *P* < 0.001)^32^.

In conclusion, in the absence of known *EGFR* mutational status, intercalating and maintenance use of gefitinib improves the PFS and OS of NSCLC patients of the adenocarcinoma subtype, or never or former light smokers who have achieved SD with chemotherapy.

## Patients and Methods

### Patients

This open-label, randomized, phase III multicenter study enrolled patients with histologically or cytologically confirmed American Joint Committee on Cancer (AJCC) non-squamous advanced NSCLC with unknown *EGFR* status who were considered unsuitable for therapy of curative intent. Main inclusion criteria were: (1) age between 18 and 75 years and never or former light smokers, defined as smoking <100 cigarettes/lifetime or abstinence from smoking for at least 15 years on the day before the start of therapy and a ≤10 pack year history; (2) Eastern Cooperative Oncology Group (ECOG) performance status of 0 or 1; 3) patients had achieved SD after two courses of first line gemcitabine plus carboplatin. Major exclusion criteria were known allergy to gefitinib, life expectancy <12 weeks, brain metastasis, and others. Patients with asymptomatic brain metastasis were included per the amended protocol.

The study was approved by the ethics committee of participating centers (Appendix I; Shanghai Chest Hospital, the First Hospital of Jilin University, Henan Cancer Hospital, the Second Affiliated Hospital of Zhejiang University School of Medicine, Jiangsu Cancer Hospital, Nanjing General Hospital, Ruijin Hospital, Huadong Hospital, the First Affiliated Hospital of Soochow University, Changhai Hospital, Hangzhou First People’s Hospital, Sir Run Run Shaw Hospital, PLA 81 Hospital, and Wuxi Fourth People’s Hospital) and performed in accordance with the Declaration of Helsinki and Good Clinical Practice guidelines. All patients provided written informed consent before participation in the study.

### Intervention

Using block randomization, eligible patients were assigned by participating physicians a randomization code and the persons who administered the medications, the raters and patients were not blind to the assignments.

Patients who had received two 4-week cycles of gemcitabine (1250 mg/m^2^) plus carboplatin (area under the curve, AUC = 5) and achieved SD were randomized to receive maximally four 4-week cycles of gemcitabine (1250 mg/m^2^) plus carboplatin (AUC = 5), with or without gefitinib (250 mg/day, on days 15–25 of each cycle) (AstraZeneca, Wilmington, DE). Patients who received gefitinib and did not progress during the maximally four cycles of chemotherapy were maintained with gefitinib (250 mg/day) after the last cycle until disease progression. Dose modifications of gemcitabine or carboplatin were based on the nadir of the absolute neutrophil or thrombocyte counts of the previous cycle.

### Patient Evaluation

Data on patient demographics, medical history including smoking history, and concurrent therapy were retrieved. All patients were evaluated monthly including ECOG performance status, routine laboratory investigations, and electrocardiography. Tumor was assessed every 56 days by computed tomography scan. The ORR included complete response (CR) and PR that were assessed using (RECIST version 1.0. PFS was calculated from the day of randomization to the first date of progression, death of any cause or the last day of follow-up. OS was calculated from the day of randomization to the last day of follow-up or death from any cause.

### Safety

Adverse events (AEs) were monitored throughout the study and graded by the CTCAE, version 3.0. Safety assessments were based mainly on the occurrence and severity of AEs. For all AEs, where necessary, patients were withdrawn from the study.

### EGFR Mutation Analysis

Archived tumor samples, which were obtained from primary or metastatic lesions after initiation of therapy, were retrospectively analyzed for *EGFR* mutations. Cytological samples were used only when histological materials were unavailable. Tumor DNA was extracted using the QIAamp DNA FFPE tissue kit (Qiagen, Crawley, UK) and *EGFR* mutation was analyzed using the amplification refractory mutation system (ARMS)-based *EGFR* mutation detection kit (Qiagen).

### Statistical Analysis

An estimated sample size of 218 patients and totally 172 events were chosen to achieve at least a statistical power of 80% to detect a difference in PFS (median PFS: 6.9 months vs. 4.5 months, hazards ratios (HR) 0.65, α = 0.05; β = 0.2) between the control and gefitinib arm with an assumed two-sided type I error of 0.05 and a dropout rate of 10%. The sample allocated ratio was 1:1.

Statistical analyses were prespecified before the database lock. The intention-to treat (ITT) population included all patients who were randomized to treatment and received at least one dose of the study drug, and had a baseline assessment and at least one post-baseline assessment. The primary endpoint was PFS and the secondary endpoints included OS, ORR, and safety. Median PFS and OS were estimated by the Kaplan-Meier method and compared by log-rank test. Treatment effects were expressed as HR and two-sided 95% confidence intervals (CI) and estimated using Cox proportional hazards model. The ORR of the ITT population was analyzed using Fisher’s exact test. PFS and OS duration was censored at the last day of follow up for patients who were still alive or whose disease did not progress.

Safety analysis mainly used descriptive statistics and included all patients who received at least one dose of the study drug and had at least one follow-up safety assessment.

All tests were two-tailed with significance set at α = 0.05. All statistical analyses were done using the SAS 9.3 software package.
